# “Being Flexible and Creative”: A Qualitative Study on Maternity Care Assistants' Experiences with Non-Western Immigrant Women

**DOI:** 10.1371/journal.pone.0091843

**Published:** 2014-03-12

**Authors:** Agatha W. Boerleider, Anneke L. Francke, Merle van de Reep, Judith Manniën, Therese A. Wiegers, Walter L. J. M. Devillé

**Affiliations:** 1 Netherlands Institute for Health Services Research (NIVEL), Utrecht, The Netherlands; 2 Department of Public and Occupational Health, EMGO Institute for Health and Care Research, VU University Medical Center, Amsterdam, The Netherlands; 3 Department of Midwifery Science, AVAG and the EMGO Institute for Health and Care Research, VU University Medical Center, Amsterdam, The Netherlands; 4 Faculty of Social and Behavioural Sciences, University of Amsterdam, Amsterdam, The Netherlands; 5 National Knowledge and Advisory Center on Migrants, Refugees and Health (Pharos), Utrecht, The Netherlands; University of Stirling, United Kingdom

## Abstract

**Background:**

Several studies conducted in developed countries have explored postnatal care professionals' experiences with non-western women. These studies reported different cultural practices, lack of knowledge of the maternity care system, communication difficulties, and the important role of the baby's grandmother as care-giver in the postnatal period. However, not much attention has been paid in existing literature to postnatal care professionals' approaches to these issues. Our main objective was to gain insight into how Dutch postnatal care providers - ‘maternity care assistants’ (MCA) - address issues encountered when providing care for non-western women.

**Methods:**

A generic qualitative research approach was used. Two researchers interviewed fifteen MCAs individually, analysing the interview material separately and then comparing and discussing their results. Analytical codes were organised into main themes and subthemes.

**Results:**

MCAs perceive caring for non-western women as interesting and challenging, but sometimes difficult too. To guarantee the health and safety of mother and baby, they have adopted flexible and creative approaches to address issues concerning traditional practices, socioeconomic status and communication. Furthermore, they employ several other strategies to establish relationships with non-western clients and their families, improve women's knowledge of the maternity care system and give health education.

**Conclusion:**

Provision of postnatal care to non-western clients may require special skills and measures. The quality of care for non-western clients might be improved by including these skills in education and retraining programmes for postnatal care providers on top of factual knowledge about traditional practices.

## Introduction

Skilled professional care is as important during the postnatal period as during pregnancy and childbirth. According to the WHO, the major goals of postnatal care are to maintain and promote the health of mother and child and to foster an environment that offers help and support to the extended family and community, for physical and mental health as well as social and cultural issues that can affect health and wellbeing. Also, postnatal care offers new parents support for parenting and the responsibilities that come with it [1].

Migration to developed countries has increased the ethnic diversity in healthcare, including postnatal care. A substantial proportion of this migration comes from developing countries where women receive less postnatal care [2]. Several studies conducted in developed countries have explored the experiences of non-western women with postnatal care, reporting both positive and negative experiences. A recurrent negative issue was the limited help received from postnatal care providers [3], [4]. Women expected support from care providers to look after the baby and allow them to rest, but this contradicted to the Western postnatal care system's ideas of self-care and mother-child bonding. Another negative issue concerned the traditional postnatal customs which are often encouraged by family members such as the mother or mother-in-law, who are very influential in the postnatal period. Some women in these studies reported a lack of knowledge about their traditional postnatal customs on the part of their care providers [4] and lack of opportunity to practice these customs [5]. Language difficulty was found to complicate communication with care providers [6], [7] which led to dissatisfaction with the care received. In a few of these studies, the negative experiences even led to women opting for early discharge from hospital [6], [7].

The perspectives of professionals who provide postnatal care to non-western women have also been explored in research, although less widely. These studies revealed different cultural practices [8], communication problems [8]–[10], and lack of knowledge of the care system [8]. However, postnatal care professionals' approach to these difficulties has received little attention in the existing literature. One study conducted in Australia describes how culturally competent midwives preserved and accommodated their Chinese and Islamic clients' cultural preferences in the postnatal period by, for example, accepting and appreciating the mother or mother-in-law as the caretaker of the baby and the new mother and heeding women's customs of keeping their hair and body covered [11]. A Dutch study conducted between 1996 and 1998 reported a list of qualities needed to provide postnatal care to Turkish and Moroccan women. Flexibility and the ability to adapt were the two of the qualities most frequently reported by the postnatal care professionals in this study [12].

There are no recent studies on Dutch postnatal care providers' approaches to difficulties with non-western women, whereas these women represent a large proportion (17%) of the total number of live births in the Netherlands [13]. Furthermore, non-western women in the Netherlands are at higher risk of adverse pregnancy outcomes such as perinatal and maternal mortality [14], [15]. They are also very diverse in origin. Turks, Moroccans, Surinamese and Antilleans/Arubans are the largest immigrant groups in the Netherlands and may have different needs and expectations for postnatal care. The Dutch postnatal care system is also quite unique. In cases of an uncomplicated childbirth, whether at home or in hospital, professional postpartum care is provided by a maternity care assistant (MCA) who attends the new mother at home for at least three hours a day during the first seven or eight days after birth [16]. MCAs are responsible for monitoring the health of mother and baby and reporting on it to the midwife. They also give instructions to the new mother (such as how to breastfeed or bathe a baby) and may do some household tasks (such as laundry) or look after the other children if time permits. In addition to the care provided by MCAs, midwives pay an average of five home visits to the new mother during the first two weeks after the birth.

Because of the large and diverse non-western client population and the unique Dutch postnatal care system, we were interested in how MCAs address the difficulties encountered in the provision of care to non-western clients. These approaches may also be useful in other developed countries with large non-western populations, as the core content of postnatal care is more or less the same across countries. We therefore conducted a study on the experiences of MCAs with non-western clients. Two questions are addressed in this article:

1) How do Dutch MCAs feel about providing care to non-western clients?

2) Do Dutch MCAs adjust their care to non-western clients and if so in what ways?

## Methods

A generic qualitative research design was used for this study. Generic qualitative research does not use one specific qualitative methodology as guidance, but does underline the theoretical positioning of the researcher, the congruence between methodology and methods, strategies to establish rigour and the analytic lens through which data are examined [17]. Individual interviews were conducted as these are suitable for obtaining rich and well explored data on the experiences and perceptions of individuals and give the opportunity to discuss sensitive topics.

### Recruitment and sample

A purposive sample of fifteen MCAs was obtained. Firstly, MCAs were recruited via employers, i.e. home care agencies or MCA agencies, located in urban areas or neighbourhoods with large non-western populations. Regional managers of these agencies were sent a letter with information about our study. They were invited to inform their MCAs providing care to non-western clients about our study and ask them if they were willing to participate. Of the nine agencies approached, six responded and brought eleven MCAs into contact with us. One of the interviewees suggested inviting another colleague as well, as this colleague had a great deal of work experience with non-western clients. This resulted in one additional interviewee. Secondly, MCAs with experience providing care to non-western women were recruited through the researchers' personal networks. This resulted in three more interviewees. All MCAs who were willing to participate received a letter with information about the study from their regional manager or the researchers. Interview appointments were made by e-mail or telephone.

Individual interviews were held with fifteen MCAs between January and March 2012. The interviewees were all women, aged between 32 and 61. Twelve were of native Dutch origin, two of non-western origin and one of western migrant origin. Their experience as an MCA ranged from one and a half to 29 years, and specifically with non-western clients from one and a half to 27 years. The MCAs differed in their client population. Most had a mixed client population which consisted of native Dutch, other western and non-western women. Two were specialised in maternity care assistance for non-Dutch women and were scheduled by their agency to provide care almost exclusively to western and non-western immigrant women. The interviews were conducted by two female researchers; nine by the first researcher (AWB) and six by the second researcher (MvdR). AWB studied medicine and public health, was trained in qualitative research methods and interviewing techniques in medical anthropology. Her interest in this topic was triggered by her own non-western background as well as her medical background. MvdR is of native Dutch origin and was trained in qualitative methodology and in-depth interviews in her medical anthropology study. Her interest in this topic arose from her master's thesis, which focused on the relationship between culture and the experience of pregnancy and childbirth in the Netherlands, as well as previous working experiences as an assistant in an urban midwifery practice.

### Interviews; data collection

A semi-structured interview guide was constructed to explore MCAs' experiences with providing care to non-western clients. This guide was reviewed by six research experts (including two midwives), and the interview questions were inspired by existing literature and the conceptual framework of Foets et al. [18], an elaboration of the Andersen model of healthcare utilisation. This conceptual framework was developed to explain ethnic differences in healthcare utilisation through various underlying factors such as migration-related characteristics, cultural factors, socioeconomic status, social network, accessibility of healthcare, personal treatment by healthcare professionals and communication. The interviewers were flexible in how the interview guide was used. The sequence of questions and the way they were asked were not predetermined and interviewees were encouraged to raise other issues not included in the guide. All relevant issues raised were added to the interview guide for subsequent interviews.

All interviews were conducted in Dutch and varied in length between 38 and 87 minutes. Eight interviews were conducted at home, six in a room at an agency, and one at a midwifery practice. They were all audiotaped. The number of interviews was not predetermined. Data saturation, the point at which the two researchers did not observe any new information or themes in the additional data, was attained after 13 interviews. Subsequently, two more interviews were conducted to confirm this. A summary was made of every interview conducted. These summaries were sent by e-mail to the interviewees, to confirm the accuracy of the summary or to suggest corrections or additions.

### Data analysis

In accordance with qualitative research principles, data collection and analysis were conducted in a cyclical process. Shortly after each interview was conducted, the full verbatim transcript was read several times by both researchers. Subsequently it was analysed thematically [19], focusing on how the MCAs approach the specific difficulties encountered among non-western women and the feelings aroused from caring for these women. To enhance the rigour, the initial analysis of each transcript was done independently by both researchers, after which they compared and discussed the main themes arising from the interview material and the related codes. The interview material was coded and ordered with the software programme MAXQDA [20]. To enhance the rigour of the analysis further, all main themes and subthemes were also discussed with the other co-authors.

### Ethics statement

Interviews were carried out as part of the national DELIVER study, which obtained approval from the medical ethics committee of the VU University Medical Center in the Netherlands (WC 008-100). All interviewees received written and verbal information about the aim and content of the interviews, the voluntariness of participation and the right to discontinue the interview or not to answer particular questions. Thereafter they gave written informed consent to be interviewed and audio recorded. The interviewers also signed the interviewees' consent form guaranteeing confidential handling of the obtained interview data.

## Results

The MCAs' experiences with non-western women were clustered into four main themes: (1) Being flexible and creative to enhance health and safety of mother and baby, describing MCAs' approaches to guarantee the health and safety of non-western women and their babies while taking their culture into account, (2) Imparting information, describing MCAs' efforts to improve non-western women's knowledge of the Dutch maternity care system, (3) Building a trusting relationship, describing the MCAs' approaches to establishing a good relationship with non-western women and their families and (4) Having predominantly positive, but also negative feelings, describing the MCAs' feelings about caring for non-western women.

### Being flexible and creative to enhance health and safety of mother and baby

Even though the MCAs encounter difficulties in the provision of care to non-western women, they all endeavoured to ensure the health and safety of mother and baby. This was achieved by being flexible towards women's preferred postnatal practices and by finding a compromise solution if these practices pose a risk. They also tried to ensure health and safety by being creative when faced with communication problems or clients with financial constraints.

#### Being flexible

The MCAs described a variety of postnatal practices among non-western women and considered most of these as innocent. However, a few such as dressing babies too warmly were considered to be harmful or dangerous. When the risk of these practices is pointed out, new mothers often seemed willing to change their behaviour. However, the MCAs also described situations where they encountered the same practice on arriving the next day, as new mothers were not able to convince their family members, mostly from the first generation, of the harm or danger. The MCAs explained that in situations where clients observe ‘different’ postnatal practices, flexibility is very important. Practices that are not consistent with the MCAs' working protocols are not rejected, as long as they pose no danger to the health and safety of mother and child. If the practice does pose a danger, the MCAs have to intervene and find a compromise solution which satisfies the mother and family, yet and at the same time does not jeopardise the health or safety of mother and baby. In situations where a compromise cannot be reached, the MCAs have no other choice but to tolerate the practice and protect themselves by recording this in their daily report and reporting it to the midwife.

I'm not a huge fan of knives in the bed as Turkish people do. And then I kindly request… I won't tell them that they have to take it out, but that it is placed on the side [sic]. Because those cots are very often large. And they often don't use cradles, but cots from the start. That helps, putting it on the other side of the bed. That. (MCA 9)

Dressing the baby. It should all be as warm as possible. Put everything on. And then we try to say, “You shouldn't do that.” And they find that annoying, because they want to do what they have learned: dressing the baby warmly because it is cold. And that of course causes conflict. And then I say, “Okay, we will dress him this way, and then we will wrap him in a blanket.” Then you have a bit of, okay, not quite, but still something. You have to find a balance. The golden mean. And if you've found it, you'll make progress. Because you're not quite with them, but you have responded to their expectations. (MCA 3)

#### Being creative

The MCAs also talked about financial constraints, which they encounter more often among non-western women than among native Dutch women. Some clients could not afford to buy the necessities for a new-born baby, and some lived in harrowing circumstances. Other clients lacked the necessities for a new-born baby because of cultural tradition. In these situations the MCAs adopt a more creative approach. By being more practical and by improvising with the scarce resources available they try to guarantee care and thus the health and safety of mother and child.

Well, what I find most distressing is that some people, for example, have no heating. So you have to boil water to fill a bath. Then I think, “It shouldn't be like that”. They are cut off from everything. Sometimes there are very serious matters. It's hard. You can do a lot with all kinds of emergency alternatives. You can use one of the mother's towels to dry the child, instead of a hydrophilic nappy. But sometimes it is very harrowing. (MCA 10)

We also need to improvise to help them. You are not going to demand, “You just have to go and buy a bathtub, because that child has to be bathed.” You're not going to do that. Then you just have to clean the sink and bathe the child in it.…If they have no cloths, you have to find an old sheet. It [the sheet] is being washed. You just improvise. (MCA 8)

The MCAs also described their efforts to improve communication with women who do not have a good command of Dutch; mainly first-generation women with origins outside the former Dutch colonies. Suitable family members and professional telephone interpreters were described as useful alternative methods of communication. However, these are not always available. In these situations the MCAs have to be creative. This means that they either use picture books or proceed to nonverbal communication, which they often called ‘talking with their hands and feet’, to demonstrate their instructions as clearly as possible. Some MCAs explained that this creative way of communication works well for aspects of postnatal care where language hardly plays a role e.g. showing mothers how to bathe or feed their babies.

The mother [mother-in-law] had been living here for 25 years, but her Dutch was poor. He [the husband] could [speak Dutch], but he was at work. And she hardly spoke Dutch and no English. That's when hands and feet are used. And we also have, which is nice, our organization is really good, we have plasticised - what do you call that? - pictograms. And then you say, “Look at this picture.” And you yourself demonstrate it. I must say, I always figure it out with hands and feet. It is sometimes quite hilarious and we have to laugh at each other. (MCA 11)

### Imparting information

Non-western women's limited knowledge of the Dutch maternity care system also came up as an important issue when caring for non-western women. The MCAs described situations where they were perceived as a guest by non-western clients. These clients tried to look after them and get them to sit down with a cup of coffee, instead of the other way around. A few MCAs described situations where they were perceived as government observers who were visiting to check how well the baby is being taken care of. In these situations, the MCAs tried to improve these women's knowledge and understanding of maternity care assistance by explaining the organization of postnatal care in the Netherlands and the tasks of an MCA. Furthermore, the MCAs described how they educated people who were following harmful or dangerous practices about the risks of those practices. Those too ashamed to be examined were educated on the importance of certain intimate physical examinations such as inspecting stitches.

A small example: in some immigrant families they tend to wrap babies very warmly. Well, that's common in their culture. But often when you start talking about the issue, you end up with a conversation about this issue in relation to sudden infant death syndrome (SIDS) in the Netherlands. Then I say, “Yes, but in the country you come from, houses are much less insulated, the houses are far too cold. Here in the Netherlands, everything is very warm, insulated, heating [sic].” And then they say, “Really?” [and then I say] “It's true.” And if you explain it clearly, they also see that the blanket can be taken off, or the cap can be taken off. (MCA 6)

And they do it because the midwife wanted them to have maternity care assistance. Well, then they register for it. Then they get the papers. But I have the idea that the people do not read. [They think:] “I have registered and will get maternity care assistance.” And they don't read further, about what maternity care assistance means. So when we come, we always ask first, “Do you know what maternity care assistance is?” Well, they don't. So then we tell them - this and that. And occasionally they are shocked, because we have to look in their cupboards. (MCA 3)

### Building a relationship with the client and the family

Building a relationship with every new client is pivotal. According to the MCAs, this requires more effort with some non-western women, especially with those who are not familiar with maternity care assistance. Nevertheless, they all tried to establish a trusting relationship in a number of ways.

#### Showing respect and interest

The MCAs explained that building a trusting relationship with every new client, irrespective of ethnic origin, starts by behaving as a guest in their house and not starting to set rules on the first day of work. This gives them the opportunity to gradually get used to each other. With non-western clients, the MCAs further establish a relationship by showing respect for their culture. Unusual customs or habits such as a husband who does not want to shake hands could easily be perceived as an offence, but they explained that these should be respected. Also, the MCAs further establish their relationship with non-western clients by showing interest and eagerness to learn more about their culture. On the other hand, the MCAs expected respect and understanding from their clients as well.

But I think that's very important. Respecting their culture. Respect gives them, I think, confidence in you. And you should also be interested in how they do things and why they do it like that. That you don't only talk about your own thing, but also show interest: “Hey, why do you do it like that?” They like to tell you about it. (MCA 14)

But also shaking hands with a man. I always wait: what does the man himself do. I say, “Hello, I'm XXX. Congratulations on your child.” And I wait to see what the man does. Does he offer to shake hands or not? Because some men absolutely refuse to. And then I think, “Yes, maybe you're also embarrassing them by offering your hand.” I wait to see what they do. I think you should have respect for all religions. Just as I think I should have the opportunity to pray before eating with a family, I also find that I must show respect towards their religion. (MCA 4)

### Involving the family

The MCAs explained that building a relationship with the client alone is not enough. In most non-western families, except for asylum seekers and refugees who often do not have extended family around, family members take over household chores and are involved in the care of the baby and other children. Besides giving support, families also exercise great influence on postnatal women by motivating them to go back to their roots and observe their traditional postnatal practices. The MCAs therefore also considered building a relationship with the client's family to be very important. By joining family activities such as lunch and providing information to family members, the MCAs also try to build a harmonious relationship with the client's family.

So getting grandma on your side is important. Not winning her over in a slimy way, but just showing that you respect grandma… Winning grandma over by showing that you respect her and that you are willing to take over their habits and rituals. To respect them. But when [harmful or dangerous] things happen, you have to show that you can draw the line: this really is not right. And you also have to be able to give proper reasons. (MCA12)

We always say that you have to win grandma over to your side. Once you've got grandmother on your side, it's totally okay. And you will notice it. If you have a good relationship with grandma, it will be fine. Then she will also want to listen to your advice. Otherwise, in no time it will all be about what they thought and how it was in their country and how they used to do things. (MCA 1)

### Having predominantly positive, but also negative feelings

The MCAs all described the ethnic diversity and the variety in postnatal customs and traditions as interesting and nice. Some MCAs even perceived it as educative. Despite these predominantly positive feelings, the MCAs do find it difficult to care for those persisting on observing harmful or dangerous practices and ignoring their advice and instructions. Furthermore, they described caring for people who do not have a good command of Dutch as difficult, intensive and sometimes even frustrating. They also felt very positively about the hospitality received from most families, which they perceived as very special. However, a few MCAs described situations where clients seemed aloof or suspicious at the start of care, or ignored them. Some MCAs did not perceive the issues in the provision of care to non-western women as difficult, but instead as motivating and challenging.

I always find it challenging to work there. Not only because of what you can learn from them, but also their customs. Always interesting. But sometimes it's very difficult to get inside, because they don't want to accept things. If they are the second generation, they may be more willing to accept, but then you still have the first generation telling them how it was done previously, or how they used to do it. And then it's hard to eliminate that. (MCA 4)

But if you are at a refugee centre, and they can only speak their own language… well, I feel sorry for those people. It touches me, because you cannot talk about their emotions… “How are you? How do you feel?” You try to do the basic things through telephone interpretation. But then you only do the most important things. You won't ask, “How do you feel at the moment? How did the birth go?” You do have the letters from the hospital, because they usually give birth at the hospital. But not the personal things. I always think they seem very lonely … I feel a bit unhappy. I would like to do much more. No, unhappy isn't the right word: frustrated. You know - I want more, I want to tell more or give more attention, and that's not possible. (MCA 14)

You have people who can really ignore you. As if you are talking to each other and they pretend you aren't standing there. You also experience that. Then they both speak the foreign language. But it can also happen, for example, that a friend is in the house, or a sister, or someone who speaks Dutch and acts as an interpreter. And then you're just a trinity [sic], the three of you together. That's very different. (MCA 1)

## Discussion

This study explored Dutch MCAs' experiences with non-western clients using a generic qualitative research approach. Data were collected by conducting individual interviews. Four main themes were found: *Being flexible and creative to enhance health and safety of mother and baby, imparting information, building a trusting relationship with the client and the family* and *having predominantly positive, but also negative feelings*.

This study adds to what is known from a previous study by Cioffi [11], which reported preservation and accommodation of non-western women's cultural preferences by midwives in the postnatal period. Our study reveals that preservation and accommodation can be accomplished by being flexible and by finding a compromise solution in cases where the health or safety of mother or child are jeopardised. In addition, this study reveals that creativity is needed to guarantee postnatal care when the necessities for taking care for a new baby are not present, regardless of whether this is due to cultural traditions or low socioeconomic status. This study also adds to what is known from the study by El Fakiri et al. [12], by showing the circumstances in which flexibility and creativity are useful. The final relevant insight from this study is the development of a relationship with the family. The above-mentioned study by Cioffi reported the family's role in taking care of the mother and child, whereas this study not only confirmed this but also highlighted the importance of developing a relationship with them. This is important, as family members - usually from the first generation – play an important role in the observance of cultural and traditional postnatal practices.

Being flexible, finding a compromise solution if necessary, being creative and gaining trust from not only the client but also the (first generation) family can allow postnatal care to be made more culturally competent and tailored to the individual needs and circumstances of non-western women. These insights are therefore relevant findings for postnatal care professionals in western countries with a large non-western client population, irrespective of the setting of care: in the hospital or at home.

The MCAs in this study responded to women's lack of knowledge of the maternity care system by providing them with information. This approach is consistent with the results of a study conducted by Priebe et al. [21], which reported explanation and education about the healthcare system by medical professionals as a good practice in healthcare for immigrants. This approach seems essential, as lack of knowledge may affect clients' expectations as well as their utilisation of care.

Language difficulties were reported to be far more pronounced among first-generation women who did not come from former Dutch colonies. It is interesting to note that the MCAs perceived communicating with women who do not have a good command of Dutch as difficult, but also thought they were able to manage well with nonverbal communication. This may be explained by the more practical nature of their tasks compared to other care professionals such as midwives who have to provide their clients with more technical information. The MCAs' approach towards these language difficulties is consistent with the results of other studies, which also reported family members, friends and professional interpreters as well as non-verbal communication as alternative methods of communication in healthcare [11], [22].

The respectful and interested attitude found in this study indicates that acceptance and understanding are prerequisites for a relationship between MCAs and non-western clients. On the one hand, all the MCAs emphasised the importance of respect for and interest in their clients' cultural traditions and practices. However on the other hand, they also expected clients to be respectful and understanding towards them. This respectful and interested attitude towards non-western women allows the MCAs to obtain a clear picture of women's needs and expectations, which is reflected in their flexible approach. As providers' cultural sensitivity has been shown to have an effect on treatment adherence [23], it may also be argued that the respectful and interested attitude may positively affect women's adherence to advice and instructions given by MCAs.

A comparison of the experiences of non-western MCAs against those of their western counterparts revealed no striking differences. The difficulties encountered and the approaches adopted by the non-western MCAs were similar to those of the other MCAs. This finding indicates that the MCAs, irrespective of origin, adjusted their care to non-western women by means of similar approaches.

### Strength and limitations

The combination of a researcher with a medical/public health background and one with a medical anthropology background can be considered an advantage, because it prevented a narrow or one-sided approach to the study.

Despite this strength, limitations need to be taken into account. Eleven of the fifteen MCAs were recruited via their maternity care agency, which may have led to selection bias, in the sense that MCAs with a more positive attitude towards non-western women might have been put forward. On the other hand, these MCAs may have more working experience with non-western women than MCAs with less positive attitudes. Because we were interested in MCAs' approaches to non-western clients, this could be considered an advantage. As there is no public register of MCAs, contacting them through their agency was the only viable way.

### Implications

The insights gained from this study result in several implications for education programmes of postnatal care providers in the Netherlands and other western countries with substantial non-western populations. This study made clear that the MCAs have all developed their own ways of coping with the difficulties encountered in caring for non-western clients. These approaches should therefore be pooled and included in education and retraining programmes as examples of ‘best practices’. Future and current MCAs will then be better prepared for working with non-western clients and their families, and may be able to provide more culturally competent care. Also, this will prevent MCAs from reinventing the wheel when caring for these clients.

Agencies could also benefit from the results of this study by including the role of the family in the guidelines for intake consultations with every new client who registers for postnatal care. By discussing the intrinsic value of an MCA to the family in these intakes, non-western clients may be better prepared about what to expect from their MCA. Finally, a study of non-western women's experiences with MCAs is suggested for future research, in order to gain a more complete picture of the interactions between MCAs and their non-western clients.

## Conclusions

The study results indicate that MCAs experience providing care to non-western clients as interesting and challenging, but sometimes difficult as well. In order to enhance the health and safety of mother and baby, MCAs have adopted flexible and creative approaches with compromise solutions where necessary. Furthermore, they have also adopted several other strategies to establish relationships with non-western clients and their families, to improve these people's knowledge of the maternity care system and to give health education. Pooling these findings and including them in education and retraining programmes for postnatal care providers may allow the quality of postnatal care to non-western women to be improved.
